# Comparative analysis on transcriptomics of ivermectin resistant and susceptible strains of *Haemonchus contortus*

**DOI:** 10.1186/s13071-022-05274-y

**Published:** 2022-05-07

**Authors:** Waresi Tuersong, Caixian Zhou, Simin Wu, Peixi Qin, Chunqun Wang, Wenda Di, Lu Liu, Hui Liu, Min Hu

**Affiliations:** 1 State Key Laboratory of Agricultural Microbiology, Key Laboratory for the Development of Veterinary Products, Ministry of Agriculture, College of Veterinary Medicine, Huazhong Agricultural University, Wuhan, 430070 Hubei China; 2 College of Animal Science and Technology, Guangxi University, Nanning, 530004 Guangxi China

**Keywords:** *Haemonchus contortus*, Ivermectin, Resistance mechanism, Transcriptome analysis

## Abstract

**Background:**

Ivermectin (IVM) is one of the most important and widely used anthelmintics in veterinary medicine. However, its efficacy is increasingly compromised by widespread resistance, and the exact mechanism of IVM resistance remains unclear for most parasitic nematodes, including *Haemonchus contortus*, a blood-sucking parasitic nematode of small ruminants.

**Methods:**

In this study, an *H. contortus* IVM-resistant strain from Zhaosu, Xinjiang, China, was isolated and assessed by the control test, faecal egg count reduction test (FECRT) and the larval development assay (LDA). Subsequently, comparative analyses on the transcriptomics of IVM-susceptible and IVM-resistant adult worms of this parasite were carried out using RNA sequencing (RNA-seq) and bioinformatics.

**Results:**

In total, 543 (416 known, 127 novel) and 359 (309 known, 50 novel) differentially expressed genes (DEGs) were identified in male and female adult worms of the resistant strain compared with those of the susceptible strain, respectively. In addition to several previously known candidate genes which were supposed to be associated with IVM resistance and whose functions were involved in receptor activity, transport, and detoxification, we found some new potential target genes, including those related to lipid metabolism, structural constituent of cuticle, and important pathways such as antigen processing and presentation, lysosome, autophagy, apoptosis, and NOD1-like receptor signalling pathways. Finally, the results of quantitative real-time polymerase chain reaction confirmed that the transcriptional profiles of selected DEGs (male: 8 genes, female: 10 genes) were consistent with those obtained by the RNA-seq.

**Conclusions:**

Our results indicate that IVM has multiple effects, including both neuromuscular and non-neuromuscular targets, and provide valuable information for further studies on the IVM resistance mechanism in *H. contortus*.

**Graphical Abstract:**

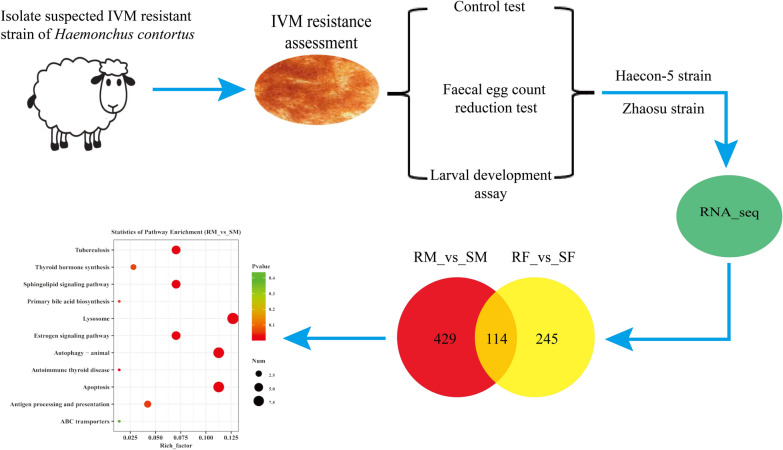

**Supplementary Information:**

The online version contains supplementary material available at 10.1186/s13071-022-05274-y.

## Background

*Haemonchus contortus* is the most prevalent and pathogenic gastrointestinal nematode (GIN) in small ruminants, causing significant economic impacts on livestock production due to its high pathogenicity and widespread occurrence around the world [1]. Owing to the lack of effective vaccines and other control measures, three classes of anthelmintics (benzimidazoles, macrocyclic lactones, and cholinergic agonists) are commonly used in the control of this parasite. Among these, ivermectin (IVM) has been successful in controlling many parasites in both humans and animals for more than 30 years [2, 3]. However, with the uncontrolled use in livestock, the success is gradually undermined by drug selection and spread of resistant parasite populations [4–6]. Consequently, resistance to IVM has become widespread in many GIN species in livestock.

Our knowledge about the resistance mechanisms of IVM has increased greatly over the past few decades. Because of technical limitations, initial research on the IVM resistance mechanism focused mainly on the polymorphisms and expression level of candidate genes coding for the target receptors, such as *Hc-lgc-37*, *Hc-glc-3*, *Hc-glc-5*, and *Hc-avr-14* [7–9]. Subsequently, attention turned to observing differences (polymorphisms and expression level) between resistant and susceptible strains or pre- and post-treatment in the P-glycoprotein-encoding genes (P-gps) and *dyf-7* [10–13]. However, due to the high levels of genetic diversity among *H. contortus* populations and the lack of consistency among studies [14–16], the major genetic mediators of IVM resistance have not been unequivocally identified.

Fortunately, the high-quality reference genome of this parasite, together with the increasing accessibility to high-throughput RNA sequencing, offers an opportunity to explore its resistance mechanism. Transcriptome sequencing has been widely used in other species to identify candidate genes/pathways related to drug resistance, such as pyrantel citrate, thiabendazole, abamectin, and IVM [17, 18]. However, the transcriptome changes in the *H. contortus* IVM-resistant field strain induced by natural selection have received little attention. In the present study, to explore transcriptomic differences and identify the candidate genes potentially related to IVM resistance in field samples induced by long-term selection, comparative transcriptomics analysis of *H. contortus* adult worms was performed between susceptible and resistant strains. The identified differentially expressed genes (DEGs) and subsequent functional analysis provided valuable information for further studies of IVM resistance in *H. contortus*.

## Methods

### IVM-resistant and IVM-susceptible strains

(1) The susceptible strain was the Haecon-5 strain kindly presented by Professor Robin B. Gasser (University of Melbourne) and maintained in goats in Huazhong Agricultural University. (2) The resistant strain was isolated from Zhaosu (Xinjiang, China) and maintained in goats in Huazhong Agricultural University.

### The control test and faecal egg count reduction test (FECRT)

In order to further confirm the IVM resistance of the Zhaosu strain, six goats (6-month-old goats free of parasites) were randomly divided into treated and untreated groups (three animals per group) and infected with infective third-stage larvae (7000 L3 per goat). The goats in the treatment group were treated with 0.2 mg/kg (with subcutaneous injection) IVM 30 days after infection. Then, all animals were necropsied 14 days post-treatment and the *H. contortus* worm burden was determined. Resistance was confirmed when the reduction in mean worm counts was less than 90%, using the formula: *R* (%) = (1 − [*E*/*C*]) × 100, where *E* and *C* represent the mean worm burden of the treated and untreated groups, respectively [19].

In addition, the FECRT was conducted based on the McMaster method [20]. After 14 days post-treatment, 2 g of individual faecal samples were homogenized in 58 ml of saturated sodium chloride. Then, 0.15 ml of the suspension was added to each chamber of the slide for egg count after 5 min. The eggs per gram (EPG) was calculated by multiplying the number of eggs in the two chambers by 100. The FEC reduction was calculated according to the formula FECR (%) = 100 × (1 − [Xt/Xc]) where Xc and Xt represent the average of EPG for untreated and treated groups, respectively. Resistance is confirmed when FECR is < 95% and the 95% confidence level is less than 90% [20].

### Larval development assay (LDA)

Fresh egg collection and LDA were carried out based on previous studies [21, 22]. Briefly, 2.4 ml suspension (2 ml egg suspension and 400 μl growth medium, ~ 5000 eggs) was added to a T25 cell bottle and incubated at 27 °C for 24 h for hatching of the first-stage larvae (L1). On the next day, the L1 suspension (99 μl, ~ 100 larvae) was aliquoted into 96-well plates and 1 μl IVM working solution was added to each well; the L1 were then incubated at 27 °C for another 6 days. The IVM concentrations ranged from 12.5 ng/ml to 0.2 ng/ml for the Zhaosu strain and 5 ng/ml to 0.1 ng/ml for the Haecon-5 strain. The number of L1 developed to L3 was counted and the L3 developmental rate was expressed as a percentage of the mean number in control wells. Three separate assays were conducted in triplicate for each concentration. The resistance ratio (RR) was calculated according to the formula: IC_50_-resistant isolates/IC_50_-susceptible isolates, where IC_50_ is the half maximal inhibitory concentration [23].

### Sample preparation

The adult male and female worms of *H. contortus* were isolated from the abomasum of goats 30 days after infection with 7000 L3 of either the Haecon-5 or Zhaosu strain. These worms were washed thoroughly in phosphate-buffered saline (PBS) and randomly assigned into four groups, each with three biological replicates including adult male worms of resistant strain (RM), adult female worms of resistant strain (RF), adult male worms of susceptible strain (SM) and adult female worms of susceptible strain (SF). All samples were transferred to liquid nitrogen for storage until use.

### RNA extraction, library preparation, and sequencing

Total RNA was extracted using TRIzol^®^ Reagent following the manufacturer’s instructions (Invitrogen, USA). RNA integrity was precisely assessed using the Agilent 2100 Bioanalyzer (Agilent Technologies, USA), and RNA samples with RNA integrity number (RIN) ≥ 8 were used to construct the library. The transcriptome libraries were prepared using an Illumina kit (San Diego, CA, USA) following the manufacturer’s instructions. Briefly, polyA messenger RNA (mRNA) was isolated using oligo (dT) beads and cleaved in fragmentation buffer. Then, complementary DNA (cDNA) was synthesized using a SuperScript double-stranded cDNA synthesis kit (Invitrogen, CA, USA) with random hexamer primers (Illumina). After quantification, the library was sequenced with the Illumina NovaSeq 6000 sequencer (Majorbio, Shanghai, China) and 150-base-pair (bp) paired-end reads were generated. The samples were named RM1, RM2, RM3; RF1, RF2, RF3; SM1, SM2, SM3; SF1, SF2, SF3, respectively. The RNA-seq raw data were submitted to the National Center for Biotechnology Information (NCBI) Sequence Read Archive with BioProject ID PRJNA772807.

### Quality control and read mapping

The sequence quality of the raw data was controlled with SeqPrep (https://github.com/jstjohn/SeqPrep) and Sickle (https://github.com/najoshi/sickle). After removing low-quality reads, the Q20, Q30, and GC content was calculated, and clean reads were mapped to the reference *H. contortus* genome (https://parasite.wormbase.org/Haemonchus_contortus_prjeb506/Info/Index/) using software HISAT2 (https://ccb.jhu.edu/software/hisat2/index.shtml) and assembled by StringTie (https://ccb.jhu.edu/software/stringtie/index.shtml?t=example).

### Differential gene expression and enrichment analysis

The transcript abundances were quantified using the RSEM software tool (http://deweylab.github.io/RSEM/) and normalized with transcripts per million reads (TPM). The DEGs were identified with |log2FC| > 1 and *P*_adj_ < 0.05 using DEseq2 (http://bioconductor.org/packages/stats/bioc/DESeq2/). The Venn, heat map clustering, and volcano diagrams were severally drawn by corresponding R packages.

To better evaluate the potential roles of the DEGs, functional analysis of the DEGs was carried out based on the Gene Ontology (GO) and Kyoto Encyclopedia of Genes and Genomes (KEGG) databases. GO and KEGG pathway enrichment analyses were performed via the Majorbio cloud platform. (https://cloud.majorbio.com/).

### Validation of transcriptome sequencing results

To confirm the validity of transcriptome data, we randomly selected 11 DEGs, including those related to lipid metabolism, cuticle morphological formation, ATP-binding cassette (ABC) transporter (P-gp), and some uncharacterized genes. The primers were designed using Primer-BLAST (Additional file 1: Table S1). cDNA was synthesized with HiScript II Q RT SuperMix (Vazyme, China), and the transcriptional level of selected DEGs was assessed in biological triplicates with technical duplicates using TB Green^®^ Premix Ex Taq™ II (Takara, Japan). The reaction procedure was as follows: 95 °C for 5 min, 40 cycles at 95 °C for 15 s, 60 °C for 15 s, and 72 °C for 15 s. The actin gene was used as an endogenous reference gene (Gene ID: DQ080917) [24], and the relative expression fold change of each gene in resistant samples versus susceptible ones was calculated using the 2^−∆∆Ct^ method.

### Statistical analysis

All experiments were independently conducted three times and analysed by GraphPad Prism 8.0.2 (GraphPad Software, USA). The *t*-test was performed to compare DEGs in resistant and susceptible strains, and the results were defined as significant for *P* < 0.05 (*P < 0.05, **P < 0.01, ***P < 0.001, ****P < 0.0001).

## Results

### The control test and FECRT

The control and FECR tests were carried out using 0.2 mg/kg of IVM in the treatment group to assess IVM efficacy. The results of the control test revealed that the percentage reduction in worm burden was 32.5% (Table 1). The results of FECRT showed that the EPG in the treatment group were not significantly decreased on day 14 post-treatment compared with the untreated group, and the FECR was 0.53% in the IVM administration group (Table 2).

**Table 1:** *Haemonchus contortus* worm burden in IVM treatment and control groups

	C1	C2	C3	E1	E2	E3
Male	1360	371	131	1069	195	160
Female	1793	473	402	1172	250	210
Total	3153	844	533	2241	445	370
Mean worm burden ± SEM	1510 ± 1169			1019 ± 865		
Reduction (%)	32.5					

*C* and *E* represent the mean worm burden of untreated and treatment groups, respectively

**Table 2:** Faecal egg count reduction rate (FECR%), 95% confidence intervals (upper CI % and lower CI %), and resistance state of *H. contortus* against IVM

	Faecal egg counts at 14 days post-treatment (EPG: eggs per gram)	
	Untreated group	Treatment group
No. 1	14,900	15,600
No. 2	1900	2200
No. 3	1800	700
Mean EPG ± SEM	6200 ± 6152	6167 ± 6698
FECR (%)		0.53
Upper 95% CI		89
Lower 95% CI		0
Drug effectiveness		Resistant

### Larval development assay

**Fig. 1:**
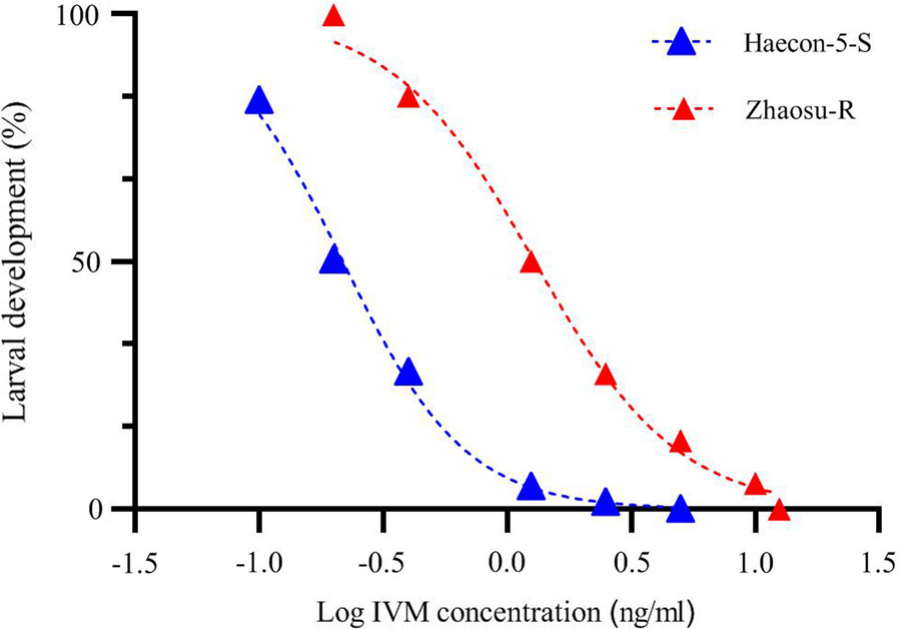
Dose responses for the Haecon-5 and Zhaosu strains of *H. contortus* towards IVM. Data for each point from three separate experiments with triplicate (mean ± SE)

The results of LDA showed that with increasing drug concentrations, the larval development rate decreased and dropped to zero at the highest IVM concentration for each group (Fig. 1). The IC_50_ values were 0.218 ng/ml (95% CI 0.208 to 0.227) and 1.291 ng/ml (95% CI 1.209 to 1.377) for Haecon-5 and Zhaosu-R, respectively. The RR was calculated as 5.9.

### Transcriptome sequencing and data assembly

**Fig. 2:**
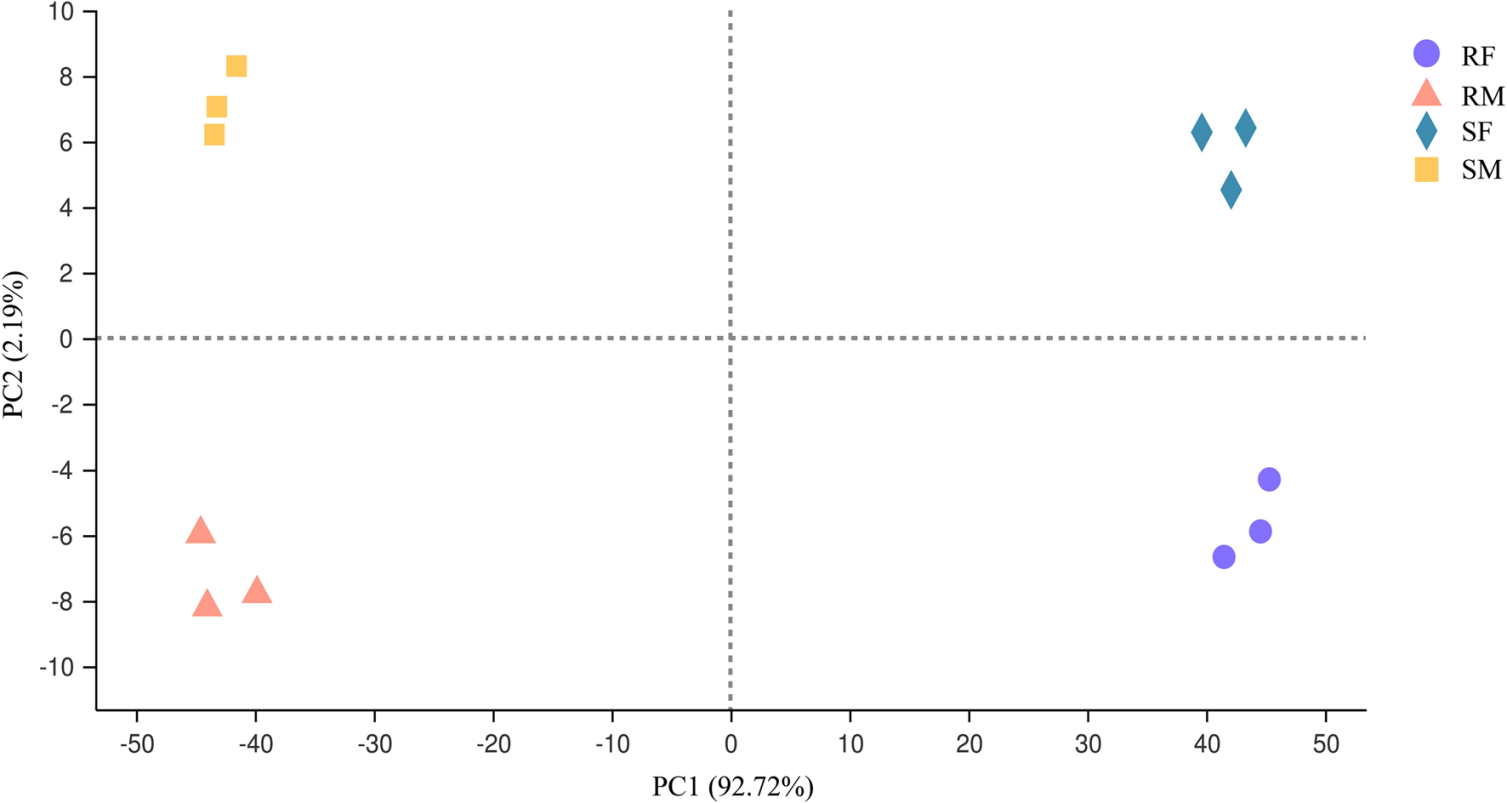
PCA of the libraries constructed from adult female and male worms of *H. contortus* IVM-susceptible and IVM-resistant strains. Purple circle, resistant female (RF); orange triangle, resistant male (RM); blue diamond, susceptible female (SF); yellow square, susceptible male (SM)

The total raw reads were generated from resistant and susceptible adult male and female worm transcriptomes (four groups) with three replicates for each sample, respectively. After quality control, a total of 93.98 GB of clean data were obtained from the 12 libraries altogether, each of which contained more than 7.19 GB, with a quality score of Q20 ≥ 98.35 and Q30 ≥ 94.71, indicating high-quality sequencing (Additional file 1: Table S2). A range of 74.6% to 78.36% clean reads of each sample were aligned onto the *H. contortus* reference genome (Additional file 1: Table S3). Principal component analysis (PCA) of the normalized RNA-seq read counts showed a high level of consistency and good separation between biological replicates of the same population from susceptible or resistant strains (Fig. 2). A total of 17,857 genes were identified and annotated; 14,083 (known: 12,898, novel: 1185), 12,153 (known: 11,266, novel: 887), 14,144 (known: 12,931, novel: 1213), and 12,405 (known: 11,487, novel: 918) genes were detected in RM, RF, SM, and SF, respectively.

### Differential gene expression analysis

**Fig. 3:**
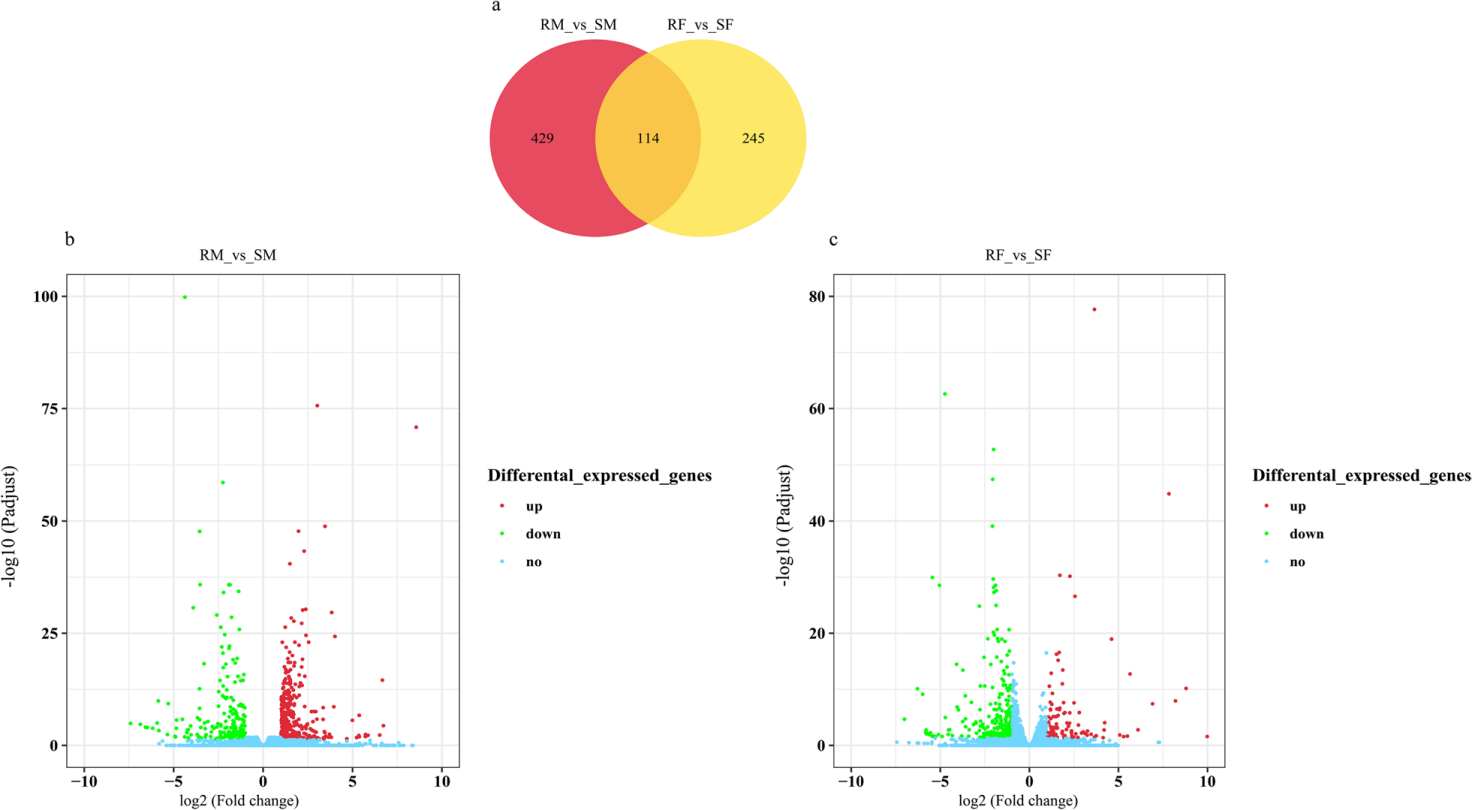
Differentially expressed gene (DEG) analysis of male and female worms between IVM-susceptible and IVM-resistant strains of *H. contortus*. **a** Venn diagram shows common and unique DEGs of the males and females. **b**, **c** Volcano plot of DEGs of males and females between IVM-susceptible and IVM-resistant strains. Red, green, and blue dots represent genes upregulated, downregulated, and without significant differences, respectively

**Fig. 4:**
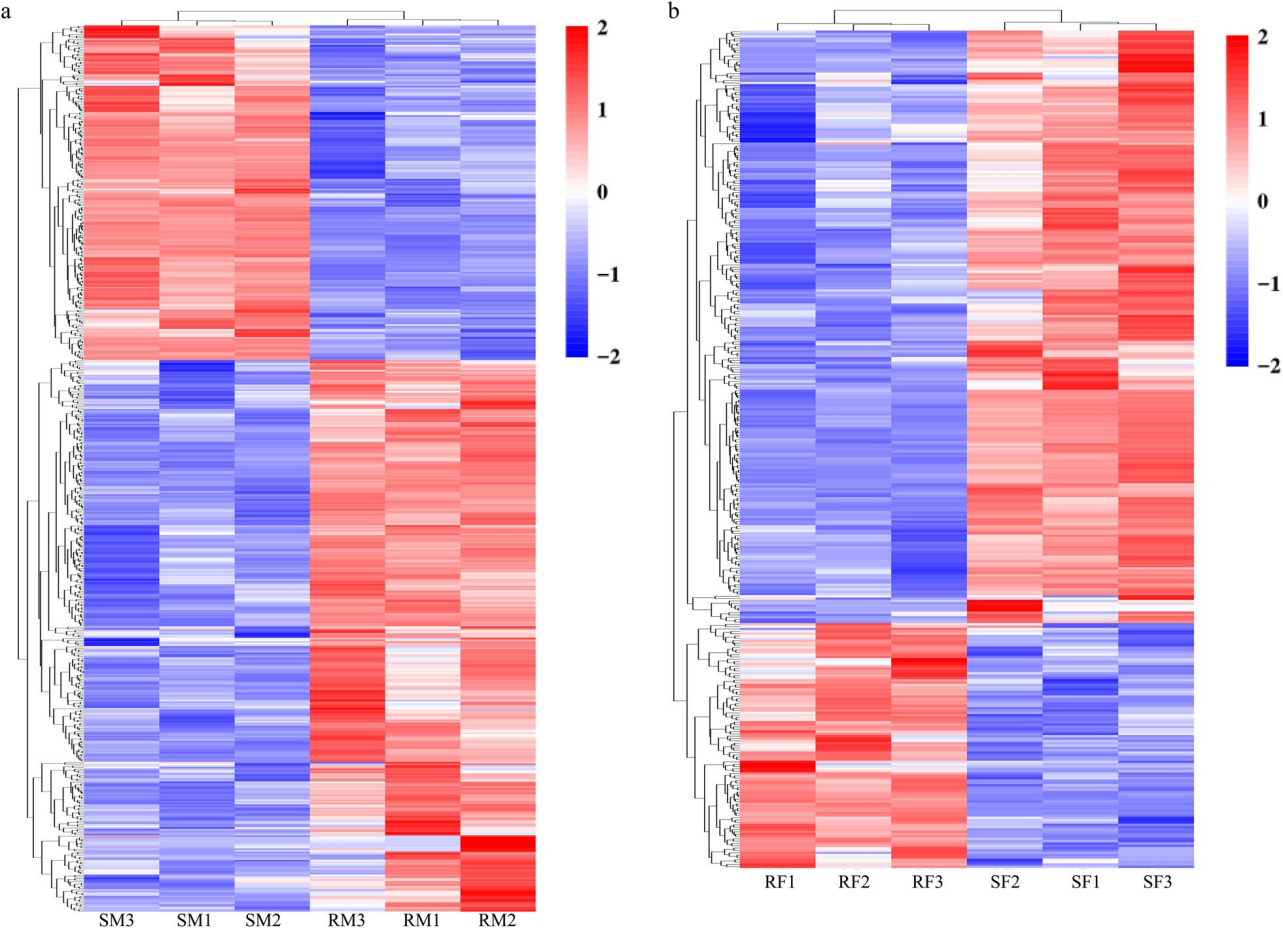
Cluster heat map showing the level of expression of genes differentially expressed in male and female worms between IVM-susceptible and IVM-resistant strains of *H. contortus*. **a**, **b** Heat map of differentially expressed genes of male and female worms between IVM-susceptible and IVM-resistant strains

In this study, DEGs were identified by comparison of the changes in transcriptome profiles of male or female worms between *H. contortus* resistant and susceptible strains. A total of 543 and 359 DEGs were identified in male (RM vs RS) and female (RF vs SF) comparisons, respectively (Fig. 3). Among them, 429 DEGs were male-specific, whereas 245 DEGs were female-specific, and 114 DEGs were present in both male and female groups (Additional file 1: Table S4, Fig. 3a). In RM, 338 genes were upregulated and only 205 downregulated, whereas in RF, 105 were upregulated and 254 downregulated. All DEGs were used for hierarchical cluster analysis (Fig. 4a, b), showing that DEGs from the biological replicates were more closely clustered together. In addition, the top 10 up- and downregulated genes in each comparison group were identified (Additional file 1: Table S5), containing some uncharacterized genes (five up and seven down for RM, two up and five down for RF) and those with known or putative functions including parasitic stage-specific protein 1, RNA-directed DNA polymerase (reverse transcriptase) domain-containing protein, saposin type B domain-containing protein, glutathione *S*-transferase (GST), SCP extracellular domain-containing protein, cytoplasmic dynein 2 light intermediate chain 1, choline ethanolamine kinase, endonuclease-reverse transcriptase, G protein-coupled receptor (GPCR), peptidase A1 domain-containing protein, and nematode cuticle collagen. In addition, DEGs with functions involved in receptor activity, transport, detoxification (Additional file 1: Table S6), lipid metabolism, and cuticle morphological formation (Additional file 1: Table S7) were also identified.

### DEGs encoding receptors

Early reports highlighted that IVM could interact with a wide range of ligand-gated channels found in parasitic nematodes, including glutamate-gated chloride ion channel receptors (GluClRs), γ-aminobutyric acid (GABA), nicotinic acetylcholine receptor (nAChR), and glycine (Gly) receptors [25]. Interestingly, among the identified DEGs, the GluClR subunit encoding gene *glc-5* (HCON_00161180) was downregulated (log2 fold-change: −4.05) in RF. In addition, the genes encoding nAChR and GPCR were dysregulated in the RM (two up, four down and five up, one down) and RF (one up, one down and one up, four down), respectively (Additional file 1: Table S6).

### DEGs involved in detoxification and transport

Except for the receptor-encoding genes, 13 genes encoding molecules involved in detoxification, including cytochromes P450 (CYPs), short-chain dehydrogenases/reductases (SDR), UDP-glycosyltransferases (UGTs), and GSTs were up- or downregulated (Additional file 1: Table S6). A total of four and two CYP450-encoding genes were found upregulated in RM and RF, respectively. Two SDR genes were only upregulated in RM and three GST genes were downregulated in both RM and RF, respectively. One UGT gene was upregulated in RM, and another one and two UGT genes were up- and downregulated in RF, respectively. In addition, the transcription level of the ABC transporter-encoding gene *P-gp-9.1* (HCON_00130050) was significantly higher in both RM and RF (log2 FC: 2.20 and 1.2, respectively), and *Hc-abt-4* (HCON_00085890, log2 FC: 1.18) was significantly upregulated in RF (Additional file 1: Table S6).

### DEGs involved in lipid metabolism and cuticle collagen formation

Beyond that mentioned above, the most notable observation is that genes related to the lipid metabolic process, cellular lipid metabolic process, lipid binding, fatty acid metabolic process, and fatty acid biosynthetic process were significantly upregulated in RM (11 genes) and RF (5 genes) (Additional file 1: Table S7). In addition, 23 and 17 cuticle collagen genes were differentially expressed in RM and RF, of which 21 (RM) and 14 (RF) were significantly downregulated (Additional file 1: Table S7).

### Functional annotation analysis of the DEGs

**Fig. 5:**
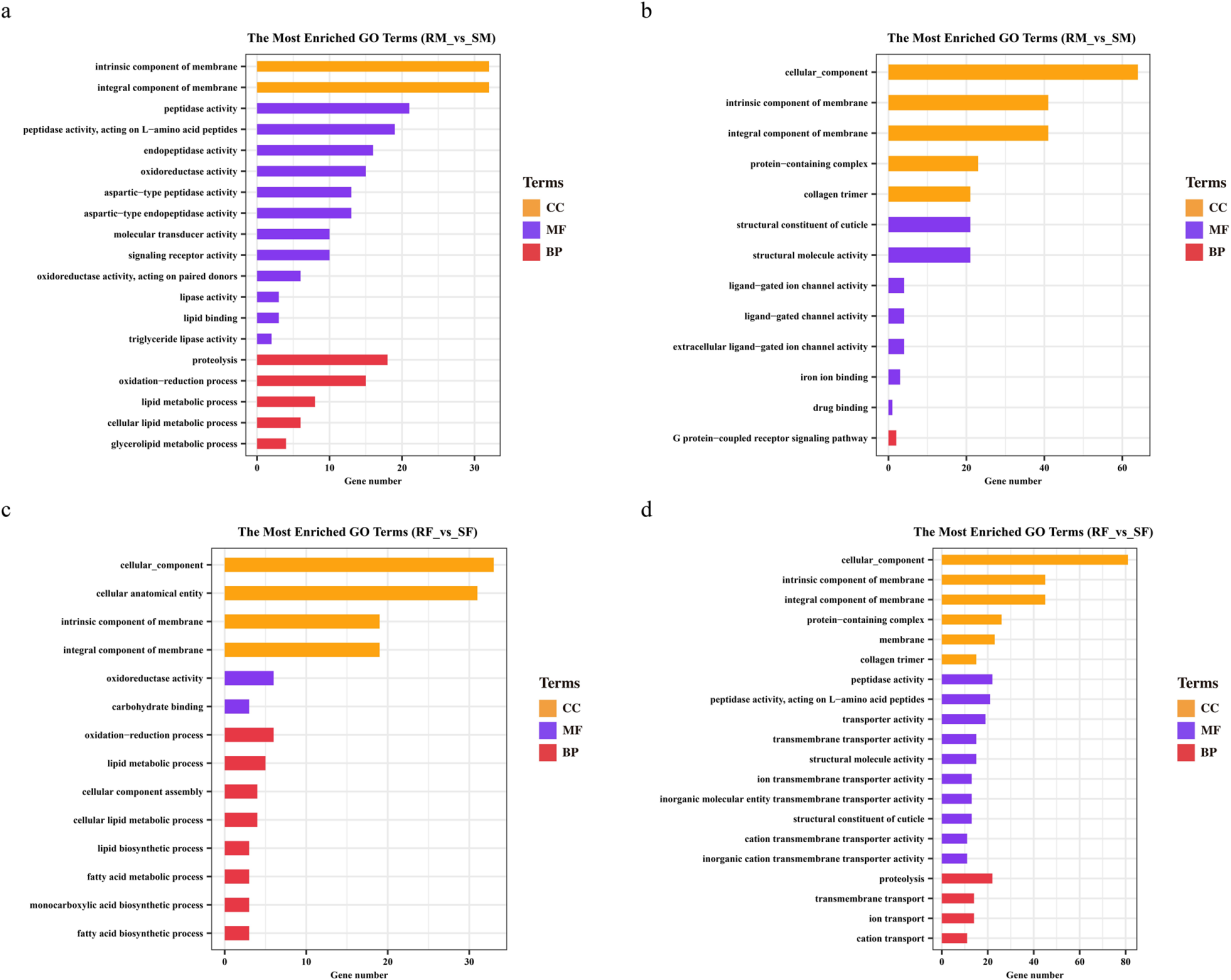
GO classifications of up- and down-regulated differentially expressed genes in male (M) and female (F) worms between IVM-susceptible (S) and IVM-resistant (R) strains of *H. contortus*. **a**, **b** GO classifications of up- and down-regulated genes of male worms between IVM-resistant and IVM-susceptible strains; **c**, **d** GO classifications of up- and down-regulated genes of female worms between IVM-resistant and IVM-susceptible strains. The *y*-axis represents GO terms; *x*-axis represents the gene number

To explore the biological functions of DEGs, the up- and downregulated genes in male and female worms were annotated with GO and KEGG. GO annotation of the upregulated genes showed some overlap between the males and females. The upregulated genes in the males and females were significantly enriched in intrinsic component of membrane (GO: 0031224), integral component of membrane (GO: 0016021), oxidoreductase activity (GO: 0016491), oxidation–reduction process (GO: 0055114), signalling receptor activity (GO: 0038023), proteolysis (GO: 0006508), and lipid metabolic process (GO: 0006508), implying that upregulated genes were mainly involved in cell integrity, signal processing, and metabolism process (Fig. 5a, c). For the downregulated genes in the males and females (Fig. 5b, d), except for terms enriched for upregulated genes, these genes were also enriched in collagen trimer (GO: 0005581), structural constituent of cuticle (GO:0042302), ligand-gated channel activity (GO:0022834), GPCR signalling pathway (GO:0007187), peptidase activity (GO:0008233), transporter activity (GO:0005215), and transmembrane transporter activity (GO:0022857), revealing that downregulated genes were also involved in cuticle integrity, transporter activity, and ion conduction process.

**Fig. 6:**
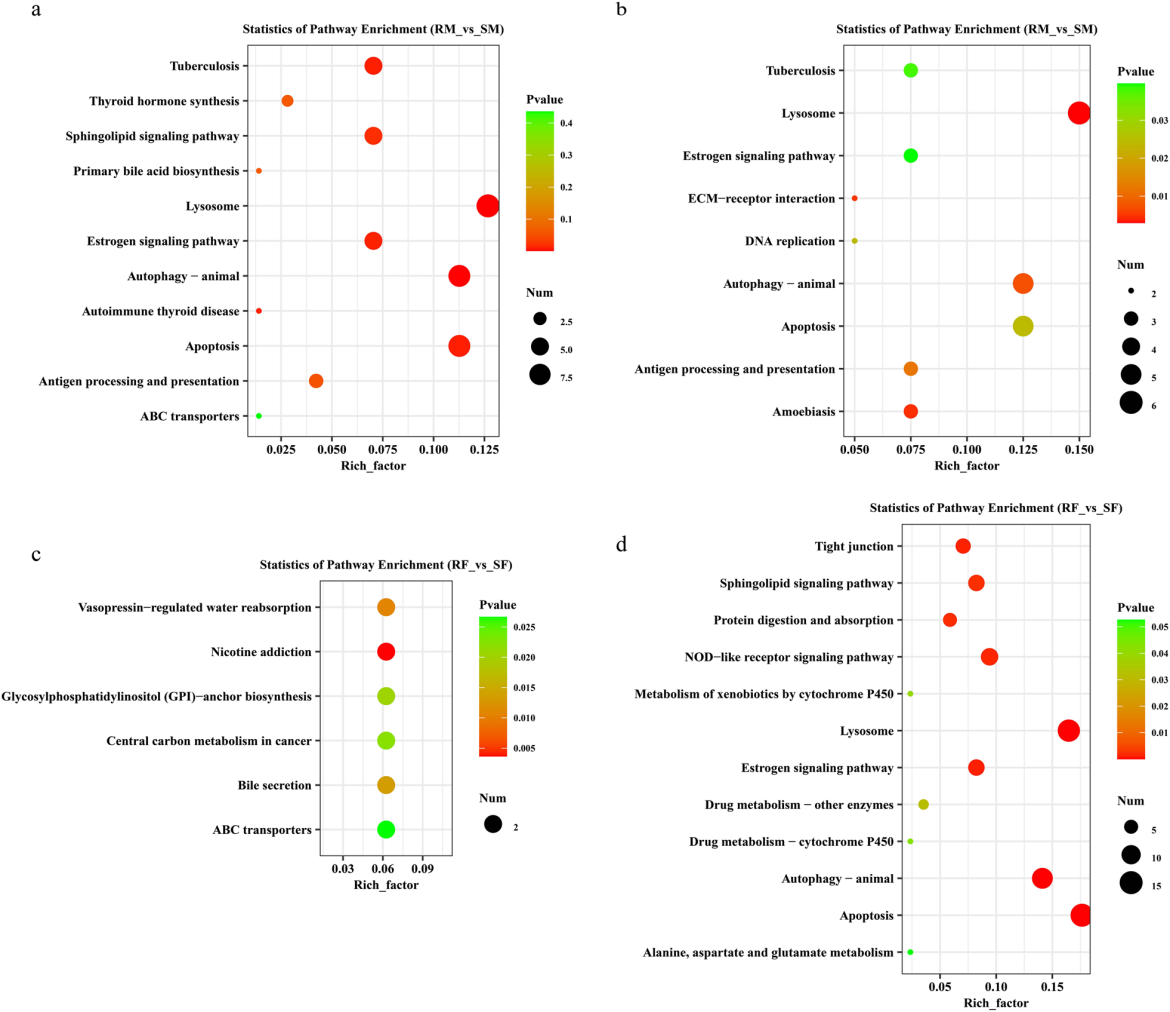
KEGG enrichment analysis of up- and downregulated differentially expressed genes (DEGs) in male and female worms between IVM-resistant and IVM-susceptible strains of *H. contortus*. **a**, **b** The most enriched KEGG pathway of up- and downregulated DEGs of male worms between IVM-resistant and IVM-susceptible strains; **c**, **d** the most enriched KEGG pathway of up- and downregulated DEGs of female worms between IVM-resistant and IVM-susceptible strains. The *x*- and *y*-axis represents rich factor and pathways. The size and colour of the circle represents the number of genes and *P*-value

The significantly enriched pathways are displayed in a scatter diagram (Fig. 6). The up- and down-regulated genes in males were significantly enriched in autophagy, lysosome, apoptosis, sphingolipid signalling pathway, and antigen processing and presentation, indicating that DEGs in males were mainly involved in metabolism, cell processes, and adaptive immunity (Fig. 6a, b). The up- and downregulated genes in females were significantly enriched in autophagy, lysosome, apoptosis, sphingolipid signalling pathway, NOD-like receptor signalling pathway, and glycosylphosphatidylinositol (GPI)-anchor biosynthesis, indicating that DEGs in females were mainly involved in metabolism, cell processes, signal processing, and protein binding (Fig. 6c, d). In addition, the ABC transporters were enriched in both males and females (upregulated).

### qRT-PCR validation

**Fig. 7:**
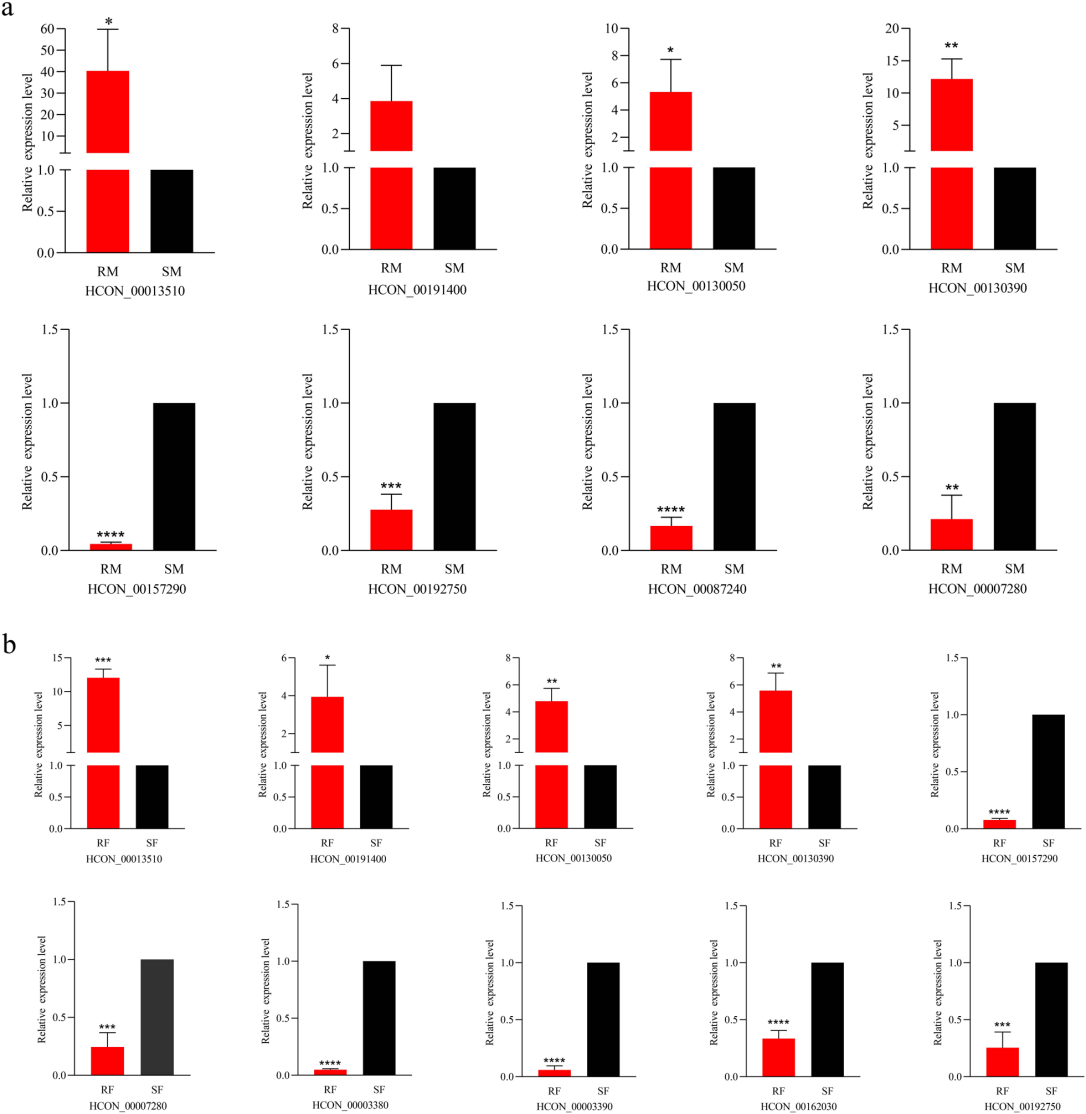
Relative transcript levels of selected genes differentially expressed in male (M) and female (F) worms between IVM-susceptible (S) and IVM-resistant (R) strains of *H. contortus*. **a**, **b** The validation of differentially expressed genes of male (**a**) and female (**b**) worms between IVM-susceptible and IVM-resistant strains. Data from the qRT-PCR represent the mean of three separate replicates, and bars represent standard error. *P < 0.05; **P < 0.01; ***P < 0.001, ****P < 0.0001

To further validate the transcriptome results, eight (male) and 10 (female) DEGs were selected for quantitative real-time polymerase chain reaction (qRT-PCR) analysis. Except for HCON_00191400 (*t*_(4)_ = 2.418, *P* = 0.0729) in RM, the results obtained were in agreement with those of RNA-seq (male, *t* test: HCON_00013510, *t*_(4)_ = 3.513, *P* = 0.0246; HCON_00130050, *t*_(4)_ = 3.136, *P* = 0.035; HCON_00130390, *t*_(4)_ = 6.222, *P* = 0.0034; HCON_00157290, *t*_(4)_ = 132.1, *P* < 0.0001; HCON_00192750, *t*_(4)_ = 11.81, *P* = 0.0003; HCON_00087240, *t*_(4)_ = 24.65, *P* < 0.0001; HCON_00007280, *t*_(4)_ = 8.365, *P* = 0.0011; female, *t*-test: HCON_00013510, *t*_(4)_ = 14.86, *P* = 0.0001; HCON_00191400, *t*_(4)_ = 3.042, *P* = 0.0383; HCON_00130050, *t*_(4)_ = 6.879, *P* = 0.0023; HCON_00130390, *t*_(4)_ = 6.138, *P* = 0.0036; HCON_00157290, *t*_(4)_ = 110.3, *P* < 0.0001; HCON_00007280, *t*_(4)_ = 10.63, *P* = 0.0004; HCON_00003380, *t*_(4)_ = 194.5, *P* < 0.0001; HCON_00003390, *t*_(4)_ = 45.31, *P* < 0.0001; HCON_00162030, *t*_(4)_ = 16.20, *P* < 0.0001; HCON_00192750, *t*_(4)_ = 9.405, *P* = 0.0007). Overall, the RNA-seq data were highly reliable (Fig. 7a, b).

## Discussion

As *H. contortus* has shown a strong ability to develop resistance, IVM resistance in this parasite from different continents has been extensively studied and well documented [26–32]. However, our understanding of the molecular basis of the resistance mechanism is patchy. Therefore, in order to further understand the mechanisms of IVM resistance in *H. contortus*, we carried out comparative transcriptome analysis between *H. contortus* adult worms of susceptible (Haecon-5) and resistant (Zhaosu) strains using RNA-seq. A total of 543 and 359 DEGs were identified in the RM versus SM (up: 338, down: 205) and RF versus SF (up: 105, down: 254), respectively. Notably, although both sexes demonstrated the same selection pressure, the number of DEGs in the RM was significantly higher than that in the RF, and more DEGs were upregulated in the RM. Considering that IVM treatment causes dysregulation in the expression of genes related to embryo development in nematodes [33], these differences might be related to the effects of IVM on the eggs in utero in female worms. Furthermore, these differences may also be related to the different mechanisms of IVM resistance between male and female worms, and the reasons hopefully can be identified in the future when more data from resistant field strains become available.

In previous studies, higher haplotype frequency and transcription-level changes in GluClR gene families such as *Hc-glc-3* and *Hc-glc-5* were often considered to be related to IVM resistance in *H. contortus* [7, 34]. Hence, it was expected that long-term selection would lead to transcriptional changes in GluClR genes in the resistant strain. As expected, the transcription level of *Hc-glc-5* was significantly reduced in RF. However, recent studies also found no significant differences in *Hc-glc-5* between IVM-resistant and IVM-sensitive isolates and no evidence of introgression in either backcross [15, 16]. Taken together, the inconsistent results from different studies were likely caused by differences in the genetic background of different strains, and suggested that *Hc-glc-5* might be related to IVM resistance in some strains, but more powerful evidence is needed to define whether *Hc-glc-5* is a major gene conferring IVM resistance.

Although IVM has often been considered to act only on different GluCl channels of nematodes, intriguingly, there is growing evidence that it also acts on other ion channels [25]. For example, in the free-living nematode *Caenorhabditis elegans*, recent studies demonstrated that IVM inhibited *C. elegans* muscle L-AChR receptors [35] and abamectin exerted more complex antagonistic effects on nAChRs [36]. In *H. contortus*, a recent study identified 26 candidate genes in relation to *H. contortus* IVM resistance, including nAChR- and GPCR-encoding genes [37]. In another study, several nAChR and GPCR genes were also up- or down-regulated in resistant strains [38]. In the present study, seven nAChR genes and 10 GPCR genes were differentially regulated in RM and RF, respectively. Among these genes, *Hc-acr-6* (HCON_0016650, predicted to be involved in cell communication, nervous system process, regulation of membrane potential, and located in synapse) was upregulated in both RM and RF, which agreed with a previous report [38]. This gene might be an important candidate gene given it is a member of ligand-gated ion channel gene families. Meanwhile, the downregulated GO terms included ligand-gated channel activity, GPCR signalling pathway, ion transport, and ion transmembrane transporter activity, implying that IVM had a significant effect on nAChRs and GPCRs. However, due to the complexity of the two receptor families, elucidating the modes of IVM action on these receptors is a considerable challenge, and further functional validation is required.

In *H. contortus*, it has been reported that up-regulation of ABC transporter-encoding genes correlates directly with the IVM resistance phenotype [39] and the final concentration of IVM in *H. contortus* [40]. In this study, *P-gp-9.1* was significantly up-regulated and the ABC transport pathway was enriched in both RM and RF (Fig. 6a, c). These results are consistent with a role for ABC transporters in IVM susceptibility in *H. contortus*, implying that ABC transporters (particularly P-gp) clearly contributed to IVM efflux in the resistant field strain.

A recent study showed that nematode defence mechanisms against xenobiotics (including endogenous compounds, anthelmintics, and environmental toxins) were dependent on the activity and overexpression of xenobiotic-metabolizing enzymes (XMEs) responsible for detoxification [41]. Therefore, some focus on the resistance mechanism turned to XMEs. In *H. contortus*, researchers have reported a potential role of XMEs in resistance to anthelmintics such as albendazole, which was oxidized in adult worms [42]. Another study showed that silencing the CYP gene (HCON_00143950), which belongs to a class of XME genes, significantly increased the sensitivity of *H. contortus* larvae to IVM [37]. In the present study, CYP genes were upregulated in both male and female worms of the resistant strain (Additional file 1: Table S6). At first sight, these upregulated CYP genes might be considered to contribute to the development of IVM resistance. However, a previous study showed that the overexpression of XMEs probably did not contribute to IVM resistance in *H. contortus*, as IVM was not metabolized by XMEs in *H. contortus* [43]. Another study showed that the predominant response was associated with an increase in lipid catabolism, as CYPs were mainly involved in lipid metabolism rather than IVM metabolism in the *C. elegans* IVM-resistant strain [44]. In the present study, up-regulated DEGs (GO) were mainly enriched in the lipid metabolic process, cellular lipid metabolic process, and fatty acid metabolic process, suggesting that XME genes may play a role in endogenous detoxification, such as lipid metabolism. Moreover, the UGT and GST genes expected to be upregulated in the resistant strain were downregulated in our study. These results further suggest that XMEs probably do not contribute to IVM resistance in *H. contortus*.

Lipids play indispensable roles in many aspects of intra- or intercellular signalling, cellular membrane integrity, and energy storage in organisms [45]. Recent studies have reported that lipids modulate the activity and expression of efflux pumps, contributing to the development of resistance in cancer [46, 47]. In nematodes, it was found that the dramatic changes after exposure to IVM were dominated by genes related to lipid metabolism (*C. elegans*) and rapid consumption of lipid stores (*Globodera pallida*) [44, 48]. Intriguingly, in the present study, up-regulated genes of the resistant strain related to lipids and lipid metabolism were enriched in GO terms, containing lipid metabolic process, fatty acid biosynthetic process, fatty acid metabolic process, lipase binding and activity, cellular lipid metabolic process, and glycerolipid metabolic process terms (Fig. 5a, c). These substantial differences in energy homeostasis between the susceptible and resistant strains suggest that IVM has multiple effects, including both neuromuscular and non-neuromuscular targets. These up-regulated genes likely play key roles in nutritional requirements, antioxidant effects, membrane fluidity, and physical rigidity of the worm cuticle, to protect the worm itself from drug stimulation and adverse environments. To sum up, lipids and their metabolic pathways may play important roles in IVM resistance, although it remains to be verified whether lipids play a role in IVM-induced stress response, or a direct role in IVM resistance.

The cuticle is an external structure in all nematodes which plays a critical role in maintaining body morphology and integrity, and locomotion [33]. Previous studies have reported that dysregulation of the collagen-encoding gene likely represents a non-specific marker of stress, such as anthelmintic exposure [33]. For example, in *B. malayi*, 17 out of 29 cuticle collagen genes were significantly downregulated after exposure to IVM [49], and in *C. elegans*, it was found that the dyf (dye-filling) mutants conferred resistance to IVM due to reduced permeability of the cuticle [50]. In addition, a recent study reported that *H. contortus* can absorb nutrients and some anthelmintics (levamisole and macrocyclic lactones) through the cuticle [51]. In the present study, 23 and 17 cuticle collagen genes displayed differential expression in both RM and RF, of which 21 and 14 were significantly down-regulated (Additional file 1: Table S7), respectively. In the GO enrichment analysis, collagen trimer and structural constituent of cuticle were significantly enriched in both RM and RF (Fig. 5b, d). These down-regulated genes play key roles in the integrity of the worm cuticle and are likely involved in protecting the worm from IVM stimulation. These results suggest that trans-cuticular penetration may be an important mode of IVM entry into worms and may represent a novel candidate pathway for IVM resistance mechanisms in the parasitic nematodes.

In recent years, considerable evidence has indicated that the lysosome, autophagy, and apoptosis pathways play important roles in cancer drug resistance [52]. Among these pathways, lysosomes contribute to the resistance of hydrophobic weak base chemotherapeutic drugs, such as sunitinib, doxorubicin, and daunorubicin, via a mechanism known as lysosomal sequestration [53–55]. Interestingly, in the present study, the lysosome pathway was one of the most significant enrichment pathways in the male and female worms. As IVM has a lipophilic nature, it may be a better-sequestered anthelmintic for lysosomes and is thus unable to reach its target sites and fails to exert its drug action.

In addition to the lysosome pathway, autophagy plays a vital role in anticancer drug resistance. A previous study showed that autophagy promotes the growth of cancer cells with multidrug resistance (MDR) and protects cancer cells from apoptosis [56]. In recent years, emerging evidence indicates that the inhibition of autophagy can facilitate MDR reversal. For example, cysteamine-elicited autophagy was found to reverse the resistance of MCF-7/ADR cells to doxorubicin, and inhibition of autophagy via genetic silencing (Atgs) sensitized MDR cells to the drug [57, 58]. Interestingly, in the present study, autophagy was also a highly significant enrichment pathway of some DEGs from both RM and RF, highlighting a potential novel biological function of autophagy in IVM resistance in *H. contortus*.

Apoptosis is an ordered and complex cellular process that strongly regulates cell death, and understanding its complex mechanism is important, as it plays a crucial role in the pathogenesis of many diseases and in resistance to anticancer drugs [59]. A previous study showed that many proteins can exert pro- or antiapoptotic activity in cancer cells, such as antiapoptotic proteins of the Bcl-2 family, and inhibition of these proteins rendered cancer cells sensitive to drug effects [60]. In the present study, apoptosis was also significantly enriched, and genes involved in this pathway were the same as those in the above two pathways. These results highlight the potential roles of these three pathways in IVM resistance in *H. contortus*.

Antigen processing and presentation and NOD-like receptor signalling pathways are important components of the immune system, which are involved in antigen processing and presentation and regulation (NOD-like receptor signalling pathway) of the immune and inflammatory responses. Previous studies reported that mutations in proteins of these pathways could also induce resistance to immunotherapies [61], and the NOD-like receptor signalling pathway was related to chemoresistance phenotypes in cancer, such as oral squamous cell carcinoma (OSCC) [62]. In the present study, antigen processing and presentation and NOD-like receptor signalling pathways were also enriched in all four groups (*P*-value greater than 0.05 was not listed in Fig. 6), suggesting that these two pathways may play a role in IVM resistance in *H. contortus*, and the exact molecular mechanisms are worthy of further exploration.

## Conclusions

In the present study, comparative analyses of the transcriptome profiles between IVM-resistant and IVM-susceptible strains of *H. contortus* were carried out. In addition to known candidate genes which were supposed to be related to IVM resistance and whose functions were associated with ligand-gated channel activity, oxidation–reduction and lipid metabolic processes, and structural constituent of cuticle, we identified many DEGs which were involved in some important pathways including antigen processing and presentation, lysosome, autophagy, apoptosis, and NOD1-like receptor signalling pathways. These findings provide valuable information for further studies on the IVM resistance mechanism in *H. contortus*.

## Supplementary Information

**Additional file 1: Table S1.** Sequence information of primers used in qPCR validation. **Table S2.** Summary of statistics for the quality control of sequencing data from adult male and female worms of ivermectin resistant and susceptible strains of *Haemonchus contortus*. **Table S3.** Summary of total reads in the transcriptome data from RNA sequencing and those mapped to the *Haemonchus contortus* genome. **Table S4.** Information on the fold changes for 114 differentially expressed genes identified in both RM_vs_SM and RF_vs_SF comparisons of *Haemonchus contortus*. **Table S5.** Top 10 up-regulated (up) and down-regulated (down) genes based on fold changes in the ivermectin (IVM)-resistant male (RM) versus IVM-susceptible male (SM) and IVM-resistant female (RF) versus IVM-susceptible female (SF) worms of *Haemonchus contortus.* **Table S6.** Information on differentially expressed genes in the ivermectin-resistant male and female worms of *Haemonchus contortus* encoding receptors, transporters, and detoxification enzymes. **Table S7.** Information on differentially expressed genes in the ivermectin-resistant male and female worms of *Haemonchus contortus* encoding molecules involved in lipid metabolism and cuticle collagen formation.

## Data Availability

The datasets of this article are included within the manuscript and its supplementary material. All the RNA-seq raw data were submitted to the National Centre for Biotechnology Information (NCBI). Sequence Read Archive with a BioProject ID: PRJNA772807.

## Abbreviations

DEGs: Differentially expressed genes
EPG: Eggs per gram
FECRT: Faecal egg count reduction test
GABA: γ-Aminobutyric acid
GIN: Gastrointestinal nematode
GluClRs: Glutamate-gated chloride ion channel receptors
GO: Gene Ontology
GPCR: G protein-coupled receptor
GPI: Glycosylphosphatidylinositol
IVM: Ivermectin
KEGG: Kyoto Encyclopedia of Genes and Genomes
LDA: Larval development assay
L1: First-stage larvae
L3: Third-stage larvae
MDR: Multidrug resistance
nAChR: Nicotinic acetylcholine receptor
NCBI: National Center for Biotechnology Information
PCA: Principal component analysis
P-gps: P-glycoprotein-encoding genes
RF: Adult female worms of resistant strain
RM: Adult male worms of resistant strain
SDR: Short-chain dehydrogenases/reductases
SF: Adult female worms of susceptible strain
SM: Adult male worms of susceptible strain
TPM: Transcripts per million reads
UGTs: UDP-glycosyltransferases
