# Supplementary material for: Comparative analysis on transcriptomics of ivermectin resistant and susceptible strains of Haemonchus contortus
Source: Parasit Vectors. 2022 May 7;15:159. doi: 10.1186/s13071-022-05274-y (PMC9077910; doi:10.1186/s13071-022-05274-y)
Supplement: Supplementary file 1 — Additional file 1: Table S1. Sequence information of primers used in qPCR validation. Table S2. Summary of statistics for the quality control of sequencing data from adult male and female worms of ivermectin resistant and susceptible strains of Haemonchus contortus. Table S3. Summary of total reads in the transcriptome data from RNA sequencing and those mapped to the Haemonchus contortus genome. Table S4. Information on the fold changes for 114 differentially expressed genes identified in both RM_vs_SM and RF_vs_SF comparisons of Haemonchus contortus. Table S5. Top 10 up-regulated (up) and down-regulated (down) genes based on fold changes in the ivermectin (IVM)-resistant male (RM) versus IVM-susceptible male (SM) and IVM-resistant female (RF) versus IVM-susceptible female (SF) worms of Haemonchus contortus. Table S6. Information on differentially expressed genes in the ivermectin-resistant male and female worms of Haemonchus contortus encoding receptors, transporters, and detoxification enzymes. Table S7. Information on differentially expressed genes in the ivermectin-resistant male and female worms of Haemonchus contortus encoding molecules involved in lipid metabolism and cuticle collagen formation. [file 13071_2022_5274_MOESM1_ESM.docx]

Table S1. Sequence information of primers used in qPCR validation

| **Primer** | **Primer Sequence 5’– 3’** |
| --- | --- |
| Actin-F | GAGTCATGGTTGGTATGGGAC |
| Actin-R | GGAGCTTCGGTCAAAAGTACG |
| HCON_00013510-F | GCGGCCCTATGGGAGATATG |
| HCON_00013510-R | TCAGCGTAGGAAGCGTGTC |
| HCON_00191400-F | CATTGCCGTGGCATCTCTTG |
| HCON_00191400-R | ACCTGCACGTCCATAATGTC |
| HCON_00130050-F | GGTATCGCAGGAGCCAATC |
| HCON_00130050-R | TGCGCGAGTGTTGTAACC |
| HCON_00130390-F | GTGTGACGCACTACAGAG |
| HCON_00130390-R | GGCCATGCTCTGTAGATAG |
| HCON_00192750-F | CGCTGGCCTTCATAGTATGTTC |
| HCON_00192750-R | GATGATACGGCTCCAGGTG |
| HCON_00087240-F | TATGTTGGCGTCGAGTGG |
| HCON_00087240-R | TCCCAAGCACCATCGGTAG |
| HCON_00007280-F | CGCCTCTTCAATGAGCAATC |
| HCON_00007280-R | GAACATGTCGGCAGTGATGG |
| HCON_00157290-F | AGTTAGTGGGTTGCGCCTAC |
| HCON_00157290-R | CGCATGAATGTGCTCCATTAGG |
| HCON_00003380-F | GTTGTACCTGTCGCATTG |
| HCON_00003380-R | GCAGGCAAGCACATGAAG |
| HCON_00003390-F | GCTTGCCCAAGGATTGGTTC |
| HCON_00003390-R | CATATTTCTGCGCCGCATC |
| HCON_00162030-F | CGCTCATCATGCCTCTATTG |
| HCON_00162030-R | AGCAGCTCCAGTCCACATC |

Table S2. Summary of statistics for the quality control of sequencing data from adult male and female worms of ivermectin resistant and susceptible strains of *Haemonchus contortus.*

| **Sample** | **Raw reads** | | **Raw bases** | **Clean reads** | **Clean bases** | **Q20(%)** | **Q30(%)** | **GC content (%)** |
| --- | --- | --- | --- | --- | --- | --- | --- | --- |
| RM-1 | | 62,181,698 | 9,389,436,398 | 61,682,312 | 9,125,257,781 | 98.43 | 94.91 | 47.61 |
| RM-2 | | 56,822,716 | 8,580,230,116 | 56,407,174 | 8,327,173,739 | 98.64 | 95.47 | 47.36 |
| RM-3 | | 50,930,596 | 7,690,519,996 | 50,603,064 | 7,515,148,399 | 98.5 | 95.09 | 47.11 |
| RF-1 | | 57,523,280 | 8,686,015,280 | 57,082,440 | 8,494,936,007 | 98.55 | 95.25 | 48.29 |
| RF-2 | | 49,714,762 | 7,506,929,062 | 49,398,626 | 7,339,071,323 | 98.62 | 95.41 | 47.56 |
| RF-3 | | 48,518,236 | 7,326,253,636 | 48,223,922 | 7,191,737,403 | 98.52 | 95.14 | 47.62 |
| SM-1 | | 51,031,890 | 7,705,815,390 | 50,587,946 | 7,530,669,870 | 98.39 | 94.87 | 47.84 |
| SM-2 | | 53,825,512 | 8,127,652,312 | 53,427,728 | 7,947,854,387 | 98.62 | 95.42 | 47.88 |
| SM-3 | | 56,694,600 | 8,560,884,600 | 56,174,962 | 8,347,339,380 | 98.48 | 95.07 | 47.89 |
| SF-1 | | 49,841,546 | 7,526,073,446 | 49,496,380 | 7,394,929,550 | 98.35 | 94.71 | 47.69 |
| SF-2 | | 49,975,174 | 7,546,251,274 | 49,672,296 | 7,359,067,621 | 98.61 | 95.38 | 47.62 |
| SF-3 | | 50,363,874 | 7,604,944,974 | 50,003,268 | 7,406,767,535 | 98.58 | 95.34 | 47.8 |

RM: male worms of ivermectin (IVM) resistant strain; RF: female worms of IVM resistant strain; SM: male worms of IVM susceptible strain; SF: female worms of IVM susceptible strain.

Table S3. Summary of total reads in the transcriptome data from RNA sequencing and those mapped to the *Haemonchus contortus* genome.

| **Sample** | **Total reads** | **Total mapped** | **Multiple mapped** | **Uniquely mapped** |
| --- | --- | --- | --- | --- |
| RM-1 | 61,682,312 | 46,982,266(76.17%) | 9,017,219(14.62%) | 37,965,047(61.55%) |
| RM-2 | 56,407,174 | 42,791,099(75.86%) | 7,876,513(13.96%) | 34,914,586(61.9%) |
| RM-3 | 50,603,064 | 38,085,707(75.26%) | 7,394,654(14.61%) | 30,691,053(60.65%) |
| RF-1 | 57,082,440 | 44,730,523(78.36%) | 4,158,596(7.29%) | 40,571,927(71.08%) |
| RF-2 | 49,398,626 | 37,710,048(76.34%) | 3,492,617(7.07%) | 34,217,431(69.27%) |
| RF-3 | 48,223,922 | 35,976,056(74.6%) | 3,166,040(6.57%) | 32,810,016(68.04%) |
| SM-1 | 50,587,946 | 38,683,461(76.47%) | 8,065,994(15.94%) | 30,617,467(60.52%) |
| SM-2 | 53,427,728 | 40,879,209(76.51%) | 8,251,713(15.44%) | 32,627,496(61.07%) |
| SM-3 | 56,174,962 | 43,112,904(76.75%) | 8,200,156(14.6%) | 34,912,748(62.15%) |
| SF-1 | 49,496,380 | 38,010,923(76.8%) | 3,743,044(7.56%) | 34,267,879(69.23%) |
| SF-2 | 49,672,296 | 37,791,042(76.08%) | 4,374,568(8.81%) | 33,416,474(67.27%) |
| SF-3 | 50,003,268 | 38,243,126(76.48%) | 4,491,629(8.98%) | 33,751,497(67.5%) |

RM: male worms of ivermectin (IVM) resistant strain; RF: female worms of IVM resistant strain; SM: male worms of IVM susceptible strain; SF: female worms of IVM susceptible strain

Table S4. Information on the fold changes for 114 differentially expressed genes identified in both RM_vs_SM and RF_vs_SF comparisons of *Haemonchus* *contortus*.

| **Gene_id** | **Log2FC(RM/SM)** | **Log2FC(RF/SF)** |
| --- | --- | --- |
| HCON_00039260 | 8.561032936 | 7.833008485 |
| HCON_00039290 | 6.66848156 | 6.91520247 |
| MSTRG.14999 | 6.520008717 | 1.012560224 |
| HCON_00119660 | 5.375615704 | 2.723921414 |
| HCON_00138380 | 4.016204595 | 2.369626065 |
| HCON_00024000 | 3.836655814 | 4.61296328 |
| HCON_00013510 | 3.461521121 | 3.653149688 |
| MSTRG.12797 | 3.357251421 | 2.365044821 |
| HCON_00137010 | 3.35206378 | 4.226744258 |
| HCON_00193760 | 3.028064089 | 1.61427141 |
| HCON_00161810 | 2.939942467 | 1.830035125 |
| HCON_00150180 | 2.897092274 | 8.795268881 |
| HCON_00016650 | 2.793348538 | 2.930339374 |
| MSTRG.11631 | 2.547967486 | 2.560034861 |
| HCON_00037300 | 2.400351239 | 1.532850873 |
| HCON_00155160 | 2.391950047 | 3.654450035 |
| HCON_00082880 | 2.388794989 | 5.648997173 |
| HCON_00191380 | 2.383020971 | 1.673149171 |
| HCON_00130390 | 2.331494883 | 2.357222318 |
| HCON_00120760 | 2.324769495 | 2.506563445 |
| MSTRG.3732 | 2.293254808 | 2.27656433 |
| HCON_00192180 | 2.213787383 | 1.851044489 |
| HCON_00130050 | 2.201137682 | 1.200033794 |
| HCON_00191400 | 2.154469952 | 1.710880227 |
| MSTRG.11617 | 2.091608459 | 1.875081635 |
| HCON_00108700 | 2.035824499 | 2.108927238 |
| HCON_00139640 | 1.921063094 | 3.129083192 |
| HCON_00024010 | 1.680186738 | 1.708527402 |
| HCON_00035360 | 1.63766449 | 1.319881476 |
| MSTRG.5094 | 1.584782293 | 1.919249236 |
| HCON_00143780 | 1.531837233 | 1.574395806 |
| MSTRG.11267 | 1.501570803 | 1.065536121 |
| MSTRG.5258 | 1.481782092 | 1.190967026 |
| HCON_00192860 | 1.480500029 | 1.292952398 |
| HCON_00137630 | 1.421119207 | 1.288735283 |
| HCON_00097850 | 1.411260201 | 1.260757771 |
| MSTRG.11628 | 1.405173612 | 1.443617587 |
| HCON_00094420 | 1.342490266 | 1.211541434 |
| HCON_00104200 | 1.333012102 | 5.500228592 |
| HCON_00124840 | 1.266644911 | 1.225877006 |
| HCON_00073950 | 1.015358623 | -1.914737845 |
| HCON_00007950 | -1.003217807 | -1.402212619 |
| HCON_00186700 | -1.071209932 | -1.112246139 |
| HCON_00076540 | -1.083304596 | -1.891891182 |
| HCON_00041920 | -1.116231911 | -1.636848662 |
| HCON_00054840 | -1.147925501 | -1.238691088 |
| HCON_00121620 | -1.194749127 | -1.02016619 |
| HCON_00162930 | -1.248806777 | -1.31456368 |
| HCON_00162030 | -1.251495768 | -2.073613482 |
| HCON_00024240 | -1.273680994 | -2.987094269 |
| MSTRG.9742 | -1.278618964 | -2.403228841 |
| HCON_00120730 | -1.286398384 | -2.334416502 |
| HCON_00107600 | -1.362763707 | -1.785015622 |
| HCON_00083430 | -1.371512358 | -1.897459768 |
| HCON_00029510 | -1.393520899 | -2.50533742 |
| HCON_00050580 | -1.4082047 | -1.239066409 |
| MSTRG.5243 | -1.432359271 | -1.157468017 |
| HCON_00161330 | -1.485943197 | -1.532735583 |
| HCON_00024700 | -1.488031605 | -1.018262002 |
| HCON_00127280 | -1.535684855 | -1.629549706 |
| HCON_00141930 | -1.538628652 | -2.019750111 |
| MSTRG.10383 | -1.572722787 | -2.064277437 |
| MSTRG.12449 | -1.578732079 | -2.329135694 |
| HCON_00081060 | -1.608381717 | -1.264521022 |
| HCON_00003650 | -1.616184872 | -1.64505281 |
| HCON_00093760 | -1.624396105 | -1.24877447 |
| HCON_00158750 | -1.626357466 | -1.271251104 |
| HCON_00038430 | -1.639235363 | -1.636483267 |
| HCON_00075990 | -1.643395155 | -1.289546322 |
| HCON_00192750 | -1.667203678 | -1.813179004 |
| HCON_00121820 | -1.681316229 | -1.427299376 |
| HCON_00057800 | -1.68934382 | -2.166673674 |
| HCON_00006360 | -1.76547835 | -1.461306762 |
| HCON_00138210 | -1.785104999 | -1.578189017 |
| HCON_00161230 | -1.830016238 | -1.789102816 |
| HCON_00032270 | -1.852632729 | -2.965698026 |
| HCON_00035990 | -1.854380983 | -2.006908152 |
| HCON_00125700 | -1.882230931 | -1.788040997 |

| HCON_00043970 | -1.898037654 | -4.718523802 |
| --- | --- | --- |
| HCON_00144340 | -1.908273097 | -1.316154541 |
| HCON_00036000 | -1.917021439 | -2.007963233 |
| MSTRG.1773 | -1.928078336 | -2.40897049 |
| MSTRG.10049 | -1.946500384 | -3.375073548 |
| MSTRG.341 | -2.080578317 | -1.89569941 |
| HCON_00156380 | -2.118090027 | -2.555681854 |
| MSTRG.9444 | -2.124268685 | -1.815719607 |
| HCON_00152640 | -2.214582129 | -1.986641102 |
| HCON_00127480 | -2.225302472 | -2.186918389 |
| HCON_00164330 | -2.232745063 | -1.665167356 |
| HCON_00142850 | -2.247944215 | -2.024909302 |
| MSTRG.12962 | -2.312248218 | -3.579583336 |
| HCON_00074200 | -2.313719589 | -1.695220483 |
| HCON_00091560 | -2.358304465 | -1.761966371 |
| HCON_00087240 | -2.367291656 | -1.369313201 |
| MSTRG.10424 | -2.372602265 | -1.547681888 |
| HCON_00076160 | -2.407481862 | -2.806998444 |
| HCON_00063040 | -2.493030631 | -3.007508238 |
| HCON_00136410 | -2.526402877 | -2.478604372 |
| MSTRG.4205 | -2.897332547 | -1.825488359 |
| MSTRG.10257 | -3.087466771 | -1.868186875 |
| HCON_00137110 | -3.299181431 | -4.474512401 |
| HCON_00007280 | -3.524228182 | -3.733892015 |
| HCON_00136420 | -3.546151648 | -2.544066447 |
| MSTRG.14874 | -3.548865975 | -6.283386146 |
| MSTRG.10264 | -3.552836302 | -1.082623962 |
| MSTRG.640 | -3.553713009 | -2.752172652 |
| HCON_00024470 | -4.177247438 | -1.421678327 |
| HCON_00157290 | -4.376745878 | -4.74208978 |
| MSTRG.4142 | -5.229592532 | -5.047051792 |
| HCON_00074860 | -5.434179412 | -2.057115866 |
| HCON_00136400 | -5.827198237 | -5.719292999 |
| HCON_00074880 | -5.859064058 | -5.444701047 |
| HCON_00074870 | -6.865344207 | -4.321560639 |
| HCON_00140540 | -7.41587251 | -5.625012006 |

Table S5. Top 10 up-regulated (up) and down-regulated (down) genes based on fold changes in the ivermectin (IVM) resistant male (RM) versus susceptible male (SM) and IVM resistant female (RF) versus susceptible female (SF) worms of *Haemonchus contortus.*

|  | **Gene ID** | **Log_2_ FC** | **Annotation** |
| --- | --- | --- | --- |
| RM-SM-up | HCON_00039260 | 8.561033 | Parasitic stage specific protein 1 |
|  | HCON_00124770 | 6.726062 | RNA-directed DNA polymerase (reverse transcriptase) domain containing protein |
|  | HCON_00039290 | 6.668482 | Saposin type B domain containing protein |
|  | MSTRG.14999 | 6.520009 | Uncharacterised |
|  | HCON_00110150 | 5.864658 | Reverse transcriptase |
|  | MSTRG.11990 | 5.760725 | Uncharacterised |
|  | MSTRG.12957 | 5.723914 | Uncharacterised |
|  | HCON_00166750 | 5.717082 | Uncharacterised |
|  | HCON_00185070 | 5.378666 | Uncharacterised |
|  | HCON_00119660 | 5.375616 | RNA-directed DNA polymerase (reverse transcriptase) domain containing protein |
| RM-SM-down | HCON_00140540 | -7.41587 | Uncharacterised |
|  | HCON_00074870 | -6.86534 | Glutathione S-transferase (hypothetical protein) |
|  | HCON_00100640 | -6.57432 | Uncharacterised |
|  | MSTRG.8420 | -6.47591 | Uncharacterised |
|  | HCON_00121230 | -6.17854 | Uncharacterised |
|  | HCON_00100600 | -5.92816 | Uncharacterised |
|  | HCON_00074880 | -5.85906 | Glutathione S-transferase (hypothetical protein) |
|  | HCON_00136400 | -5.8272 | Uncharacterised |
|  | HCON_00074860 | -5.43418 | Glutathione S-transferase |
|  | HCON_00027510 | -5.35102 | Uncharacterised |
| RF-SF-up | HCON_00140230 | 9.981274 | SCP extracellular domain containing protein |
|  | HCON_00150180 | 8.795269 | Cytoplasmic dynein 2 light intermediate chain 1 |
|  | MSTRG.4016 | 8.199537 | Uncharacterised |
|  | HCON_00039260 | 7.833008 | Parasitic stage specific protein 1 |
|  | HCON_00039290 | 6.915202 | Saposin type B domain containing protein |
|  | MSTRG.5671 | 6.096023 | Uncharacterised |
|  | HCON_00082880 | 5.648997 | Nematode cuticle collagen |
|  | HCON_00104200 | 5.500229 | Choline ethanolamine kinase and UDP-glucuronosyl |
|  | HCON_00001960 | 5.291722 | Endonuclease-reverse transcriptase |
|  | MSTRG.3948 | 5.072978 | GPCR |
| RF-SF-down | MSTRG.732 | -7.01045 | Nematode cuticle collagen |
|  | MSTRG.14874 | -6.28339 | Uncharacterised |
|  | HCON_00012250 | -5.98918 | Peptidase A1 domain containing protein |
|  | MSTRG.6402 | -5.81638 | Myosin head |
|  | HCON_00066450 | -5.79422 | Retrotransposon and Integrase domain containing protein |
|  | HCON_00066440 | -5.77946 | Uncharacterised |
|  | HCON_00136400 | -5.71929 | Uncharacterised |
|  | HCON_00140540 | -5.62501 | Uncharacterised |
|  | HCON_00152810 | -5.54436 | Hypothetical protein CBG24224 |
|  | HCON_00023610 | -5.45101 | Uncharacterised |

Table S6. Information on DEGs in the ivermectin resistant male and female worms of *Haemonchus contortus* encoding receptors, transporters, detoxification enzymes.

| **Classification** | **Gene ID** | **Log_2_ FC** | | **Annotation** |
| --- | --- | --- | --- | --- |
|  |  | **Male** | **Female** |  |
| Receptor | HCON_00161180 |  | -4.056 | Glutamate-gated chloride channel |
|  | HCON_00041490 | 1.726 |  | Nicotinic acetylcholine receptors |
|  | HCON_00016650 | 2.793 | 2.93 | Nicotinic acetylcholine receptors |
|  | HCON_00003530 | -1.037 |  | Nicotinic acetylcholine receptors |
|  | HCON_00137150 | -1.174 |  | Nicotinic acetylcholine receptors |
|  | MSTRG.12918 | -1.830 |  | Nicotinic acetylcholine receptors |
|  | HCON_00097440 | -2.590 |  | Nicotinic acetylcholine receptors |
|  | HCON_00003560 |  | -1.134 | Nicotinic acetylcholine receptors |
|  | HCON_00162230 | 1.299 |  | GPCR domain containing protein |
|  | HCON_00140940 | 1.422 |  | GPCR domain containing protein |
|  | HCON_00069180 | 1.505 |  | GPCR domain containing protein |
|  | MSTRG.10539 | 1.791 |  | GPCR domain containing protein |
|  | HCON_00124980 | 2.004 |  | GPCR domain containing protein |
|  | HCON_00145330 |  | 1.541 | GPCR domain containing protein |
|  | HCON_00121620 | -1.194 | -1.02 | GPCR domain containing protein |
|  | HCON_00110140 |  | -1.254 | GPCR domain containing protein |
|  | HCON_00156550 |  | -1.544 | GPCR domain containing protein |
|  | HCON_00162320 |  | -4.537 | GPCR domain containing protein |
| Transport | HCON_00130050 | 2.201 | 1.2 | ABC transporter |
|  | HCON_00085890 |  | 1.183 | ABC transporter |
| Detoxification | HCON_00024000 | 3.836 | 4.612 | Cytochrome P450 |
|  | HCON_00024010 | 1.68 | 1.708 | Cytochrome P450 |
|  | HCON_00141020 | 1.502 |  | Cytochrome P450 |
|  | HCON_00141050 | 1.409 |  | Cytochrome P450 |
|  | HCON_00059240 | 2.397 |  | Short-chain dehydrogenases/reductases (SDR) |
|  | HCON_00102400 | 1.154 |  | Short-chain dehydrogenases/reductases (SDR) |
|  | HCON_00127680 | 2.018 |  | UDP-glycosyltransferases (UGTs) |
|  | HCON_00108700 |  | 2.1 | UDP-glycosyltransferases (UGTs) |
|  | HCON_00125550 |  | -1.073 | UDP-glycosyltransferases (UGTs) |
|  | HCON_00125530 |  | -2.490 | UDP-glycosyltransferases (UGTs) |
|  | HCON_00074860 | -5.434 | -2.057 | Glutathione S-transferase (GSTs) |
|  | HCON_00074880 | -5.859 | -5.444 | Glutathione S-transferase (GSTs) |
|  | HCON_00074870 | -6.865 | -4.321 | Glutathione S-transferase (GSTs) |

Table S7. Information on DEGs in the ivermectin resistant male and female worms of *Haemonchus contortus* encoding molecules involved in lipid metabolism and cuticle morphological formation.

| **Classification** | **Gene ID** | **Log_2_ FC** | | **Annotation** |
| --- | --- | --- | --- | --- |
|  |  | **Male** | **Female** |  |
| lipids | HCON_00039290 | 6.668 | 6.915 | lipid metabolic process |
|  | HCON_00105450 |  | 4.195 |  |
|  | HCON_00130390 | 2.331 | 2.357 |  |
|  | HCON_00121910 | 4.992 |  |  |
|  | MSTRG.10192 | 2.82 |  |  |
|  | HCON_00093190 | 1.496 |  |  |
|  | HCON_00125510 | 1.376 |  |  |
|  | HCON_00080850 | 1.132 |  |  |
|  | HCON_00188210 | 1.116 |  |  |
|  | HCON_00120760 | 2.324 | 2.506 | Lipid transport and metabolism |
|  | HCON_00140140 |  | 1. 213 | fatty acid biosynthetic process |
|  | MSTRG.5004 | 1.718 |  | lipase activity |
|  | HCON_00139000 | 1.095 |  | lipid binding |
| Cuticle collagen | HCON_00082880 | 2.388 | 5.64 | Nematode cuticle collagen |
|  | HCON_00191400 | 2.154 | 1.71 |  |
|  | HCON_00050600 |  | 1.06 |  |
|  | HCON_00182130 | -1.01 |  |  |
|  | HCON_00192150 | -1.08 |  |  |
|  | MSTRG.12394 | -1.39 |  |  |
|  | HCON_00050580 | -1.4 | -1.23 |  |
|  | HCON_00192750 | -1.66 | -1.81 |  |
|  | MSTRG.9687 | -1.72 |  |  |
|  | HCON_00191700 | -1.81 |  |  |
|  | HCON_00100700 | -2.03 |  |  |
|  | MSTRG.9444 | -2.12 | -1.81 |  |
|  | HCON_00087240 | -2.36 | -1.36 |  |
|  | MSTRG.10570 | -2.37 |  |  |
|  | MSTRG.10424 | -2.37 | -1.54 |  |
|  | MSTRG.4205 | -2.89 | -1.82 |  |
|  | MSTRG.10257 | -3.08 | -1.86 |  |
|  | MSTRG.7115 | -3.27 |  |  |
|  | MSTRG.10264 | -3.55 | -1.08 |  |
|  | MSTRG.640 | -3.55 | -2.75 |  |
|  | MSTRG.1965 | -4.02 |  |  |
|  | MSTRG.6901 | -4.05 |  |  |
|  | MSTRG.9582 | -4.23 |  |  |
|  | MSTRG.4142 | -5.22 | -5.04 |  |
|  | HCON_00192760 |  | -1.18 |  |
|  | MSTRG.9727 |  | -1.58 |  |
|  | HCON_00003660 |  | -2.26 |  |
|  | MSTRG.732 |  | -7.01 |  |
